# *In vitro* and *ex vivo* evaluation of tumor-derived exosome-induced dendritic cell dysfunction in mouse

**DOI:** 10.1016/j.xpro.2021.100361

**Published:** 2021-03-04

**Authors:** Wenfeng Zeng, Xiaozhe Yin, Yunhan Jiang, Lingtao Jin, Wei Liang

**Affiliations:** 1 Protein and Peptide Pharmaceutical Laboratory, Institute of Biophysics, Chinese Academy of Sciences, 15 Datun Road Chaoyang District, Beijing 100101, China; 2 University of Chinese Academy of Sciences, Beijing 100864, China; 3 Department of Anatomy and Cell Biology, College of Medicine, University of Florida, 2033 Mowry Road, Gainesville, FL 32610-3033, USA

**Keywords:** Cancer, Cell-based assays, Flow cytometry/mass cytometry, Immunology

## Abstract

Exosomes that contain various signaling molecules, such as proteins, nucleotides, metabolites, and lipids, are important for intercellular communication. Dendritic cells (DC) are central regulators of anti-tumor immunity but can be suppressed by tumor-derived exosomes (TDEs) in the tumor microenvironment. Here, we describe a step-by-step protocol for TDE isolation and evaluation of TDEs on DCs both *in vitro* and *ex vivo* with high repeatability. This approach is useful for the interrogating TDE-DC interactions and identification of novel immune regulators.

For complete details on the use and execution of this protocol, please refer to Yin et al. (2020).

## Highlights

• Separation of tumor-derived exosomes (TDEs) from tumor cell supernatant
• Generation of TDE-treated DCs *in vitro* and *ex vivo*
• Magnetic purification of CD8^+^ T cell from OT I transgenic mice spleen
• Analysis of T cell proliferation via flow cytometry

## Graphical abstract

**Figure:**
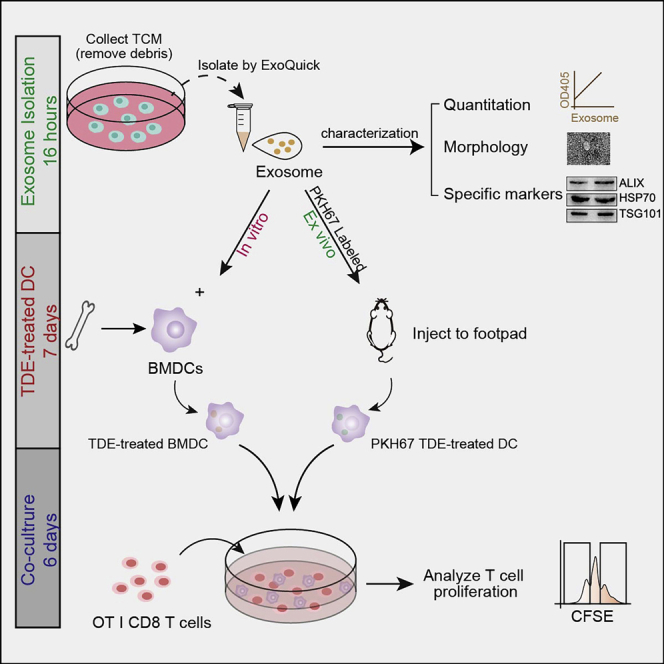

## Before you begin

### Exosome-depleted fetal bovine serum preparation

**Timing: 2 h**

Exosome-depleted FBS is prepared by centrifuging at 100,000 × *g* for 2 h at 4°C, and supernatant are collected for future RPMI 1640 medium preparation.

***Note:*** To exclude the potential effect of FBS-related exosome, exosome-depleted FBS is required for all exosome-isolating experiments.

### Anesthesia preparation

**Timing: 30 min**

Prepare 20 mL tribromo-ethanol anesthesia.

1. Weigh 0.25 g 2,2,2-tribromo-ethanol and dissolve in 0.5 mL 2-methyl-2-butanol, by vortex.
2. Heat 19.5 mL ddH_2_O to 50°C.
3. Mix 1 and 2 thoroughly, and heat at 50°C for 20 min.
4. Sterilize the solution with 0.22 μm filter and store at 4°C for up to 6 months.

### Antibody mix preparation

**Timing: 1 h**

Prepare the antibody mix before staining the sample.

1. Antibody mix for BMDC as the flow panel below.

**Table:** 

Fluorophore	Marker	Clone	Final dilution	Volume
APC	CD11c	N418	1/100	1 μL
FITC	MHC II	M5/114.15.2	1/200	0.5 μL

2. Antibody mix for OT I CD8^+^ T cell as the flow panel below.

**Table:** 

Fluorophore	Marker	Clone	Final dilution	Volume
PE	TCRVa2	B20.1	1/100	1 μL
APC	CD8a	53–6.7	1/100	1 μL

3. Antibody mix for DCs from lymph node as the flow panel below.

**Table:** 

Fluorophore	Marker	Clone	Final dilution	Volume
BV605	CD45	30-F11	1/200	0.5 μL
PE	CD11b	M1/70	1/400	0.25 μL
PerCP/Cyanine5.5	Ly6C	HK1.4	1/200	0.5 μL
PE/Cy7	MHC II	M5/114.15.2	1/400	0.25 μL
BV785	F4/80	BM8	1/200	0.5 μL
APC/Cy7	CD11c	N418	1/100	1 μL

4. Antibody mix for proliferation of OT I CD8^+^ T cell as the flow panel below.

**Table:** 

Fluorophore	Marker	Clone	Final dilution	Volume
PE	CD11c	N418	1/100	1 μL
APC	CD8a	53–6.7	1/100	1 μL
PE/Cy7	CD3	17A2	1/200	0.5 μL

## Key resources table

**Table:** 

REAGENT or RESOURCE	SOURCE	IDENTIFIER	
**Antibodies**			

Anti-mouse CD8a APC	BioLegend	Cat # 100712; RRID: AB_312751	
Anti-mouse CD11b PE	eBioscience	Cat # 12-0112-82; RRID: AB_2734869	
Anti-mouse CD11c APC/Cy7	BioLegend	Cat # 117323; RRID: AB_830646	
Anti-mouse CD11c APC	BioLegend	Cat # 117310; RRID: AB_313779	
Anti-mouse CD11c PE	BioLegend	Cat # 117307; RRID: AB_313776	
Anti-mouse CD3 PE/Cy7	BioLegend	Cat # 100219; RRID: AB_1732068	
Anti-mouse F4/80 BV785	BioLegend	Cat # 123141; RRID: AB_2563667	
Anti-mouse MHC II PE/Cy7	BioLegend	Cat # 107630; RRID: AB_2069376	
Anti-mouse MHC II FITC	BioLegend	Cat # 107615; RRID: AB_493524	
Anti-mouse CD45 BV605	BioLegend	Cat # 103139; RRID: AB_2562341	
Anti-mouse Ly6C PerCP/Cyanine5.5	BioLegend	Cat # 128012; RRID: AB_1659241	
Anti-mouse-TCRVα2 PE	BioLegend	Cat # 127809; RRID: AB_1089251	
TruStain FcXTM PLUS (anti-mouse CD16/32) Antibody	BioLegend	Cat # 156603; RRID: AB_2783137	

**Chemicals, peptides, and recombinant proteins**			

Ovalbumin from Egg White	BBI	Cat # A003056-0100	
PKH67 Green Fluorescent Cell Linker Midi Kit	Sigma	Cat # MIDI67-1KT	
CFSE	eBioscience	Cat # 65-0850-84	
Recombinant murine GM-CSF	Peprotech	Cat # 315-03	
Recombinant murine IL-4	Peprotech	Cat # 214-14	
Bovine serum albumin (BSA)	Millipore	Cat # A3733	
b-Mercaptoethanol	Gibco	Cat # 21985032	
2,2,2-Tribromo-ethanol	Sigma	Cat # T48402	
2-Methyl-2-butanol	Sigma	Cat # 152463	
Pierce ECL western blotting substrate	Thermo Fisher	Cat # 32106	
Protease inhibitor cocktail	Sigma	Cat # P8340-1ML	

**Critical commercial assays**			

MojoSort Mouse CD8 T cell Isolation Kit	BioLegend	Cat # 480035	
Mojo buffer	BioLegend	Cat # 480017	
10× RBC lysis buffer	eBioscience	Cat # 00-4300-54	
ExoAb Antibody Kit	SBI	Cat # EXOAB-KIT-1	
ExoQuick-TC	SBI	Cat # EXOTC50A-1	
EXOCET Exosome Quantitation Kit	SBI	Cat # EXOCET96A-1	
BCA Protein Assay Kit	Pierce	Cat # 23225	

**Experimental models: cell lines**			

MC38 lines	Laboratory of Yangxin Fu	N/A	

**Experimental models: organisms/strains**			

C57BL/6	Vital River Laboratory Animal Technology	Cat # 213	
C57BL/6-Tg (TcraTcrb)1100Mjb/J	The Jackson Laboratory	Cat # N000208	

**Software and algorithms**			

FlowJo	BD	https://www.flowjo.com/solutions/flowjo; RRID: SCR_008520	
GraphPad Prism 8	GraphPad Software	https://www.graphpad.com:443; RRID: SCR_002798	
ImageJ	ImageJ public freeware	https://imagej.nih.gov/nih-image/index.html; RRID: SCR_003073	

**Other**			

Amicon Ultra-2 (100 kDa MWCO)	Merck Millipore	Cat # UFC910024	
70 μm cell strainers	Falcon	Cat # 352350	
Optima XPN-100 ultracentrifuge	Backman Coulter	Cat # A99846	
Allegra X-22R centrifuge	Backman Coulter	Cat # 392188	
Zetasizer Nano ZS	Malvern Instruments	N/A	

## Materials and subject details

### 1× RIPA buffer

For 100 mL 1×RIPA buffer:

**Table:** 

Reagent	Final Concentration	Add to 100 mL
Tri-HCl pH 7.6	25 mM	302.85 mg
NaCl	150 mM	876.6 mg
NP-40	1%	1 mg
Sodium deoxycholate	1%	1 mg
SDS	1%	1 mg

Stored at −20°C for up to 3 months. Add protease inhibitors before use.

### 1× TBS buffer

For 1,000 mL 1×TBS buffer:

**Table:** 

Reagent	Final Concentration	Add to 1,000 mL
Tri-HCl pH 7.4	20 mM	2.4 g
NaCl	150 mM	8.7 g
Distilled H_2_O	n/a	Up to 1 L, pH adjusts to 7.6

Stored at 25°C for up to 3 months.

### ACK lysis buffer

For 1 L 10×ACK lysis buffer:

**Table:** 

Reagent	Final Concentration	Add to 1 L
NH_4_Cl	1.55 M	82.9 g
KHCO_3_	100 mM	10 g
Na_2_-EDTA	0.5 M	0.372 g
NaOH 1 M	n/a	Adjust pH to 7.2
Distilled H_2_O	n/a	Up to 1 L

Stored at 4°C for up to 3 months. Dilute 10 times with ddH_2_O before use.

### CFSE solution

To make 10 mM CFSE stock, dissolve 500 μg CFSE powder in 90 μL DMSO, store at −20°C for up to 6 months. For 10 μM CFSE working solution, add 1 μL CFSE stock into 1 mL PBS, store at 25°C, avoid light, and use immediately.

### BSA solution

To make 10% BSA solution, dissolve 2 mg BSA powder in 20 mL ddH_2_O, Sterilize the solution with 0.22 μm filter and store at 4°C for up to 6 months.

### FACS buffer

To prepare 100 mL FACS buffer, dilute 2 mL FBS (final concentration 2%, v/v) in 100 mL 1× PBS, mix well and store at 4°C for up to 1 month.

### Fc blocker buffer

To prepare Fc blocking buffer for each sample, add 0.5 μL of anti-mouse CD16/32 antibody (Key resources table) in 50 μL FACS buffer. Always prepare before use.

### Animals

Female C57BL/6 mice (6–8 weeks old) were purchased from Vital River Laboratory Animal Technology (Beijing, China). OT-I T cell receptor-transgenic mice (6–8 weeks old, male or female) (C57BL/6-Tg (TcraTcrb)1100mjb) whose T cell receptors recognize ovalbumin (OVA) residues 257–264 in the context of H2Kb were obtained from the Jackson Laboratory (Bar Harbor, ME, USA). All animal experiments were performed according to the institutional ethical guidelines on animal care and the protocols used for this study were approved by the Animal Care and Use Committee at the Institute of Biophysics, Chinese Academy of Sciences.

## Step-by-step method details

### Isolation and characterization of TDEs

**Timing: 16 h**

**Figure 1:**
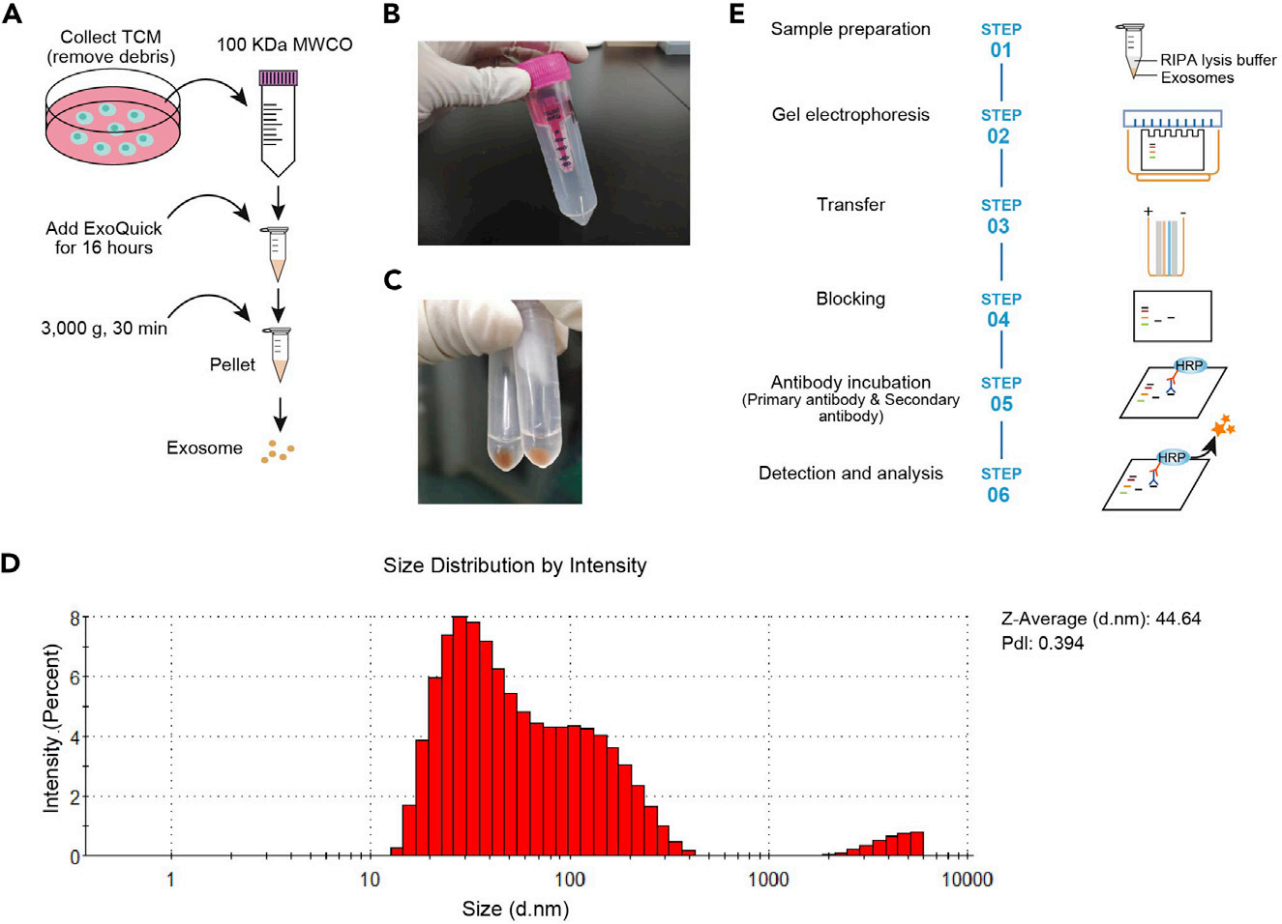
Isolation of exosomes from tumor culture medium (A) Illustrative overview of exosome separation. (B) 100 kDa MWCO to separate > 100 kDa fraction from tumor culture medium. (C) The exosome pellet is brown in appearance. (D) Dynamic light scattering analysis of exosomes. PdI, polydispersity index. (E) Western blot workflow.

This step details how to isolate TDEs from tumor culture medium (TCM) (Figure 1A).

1. Culture tumor cells in RPMI 1640 media supplemented with 10% exosome-depleted FBS. Collect supernatant of tumor cells after 48 h when cells reach 80% confluency.

***Note:*** In this protocol, MC38 mouse colon cancer cell line is used.

2. Remove cell debris from tumor culture medium (typically 20 mL) by centrifuging at 3,000 × *g* for 2 h at 4°C. Then transfer supernatant to a sterile 100 kDa MWCO (Molecular Weight Cut Off) (Figure 1B), and centrifuge at 3,000 × *g* for 30 min at 4°C to discard the faction < 100 kDa, and finally make the concentrated-supernatant (usually 1.5 mL).

**Pause point:** Concentrated-supernatant can be stored at −80°C for up to 6 months until future use.

***Note:*** An additional 10,000 × *g* centrifugation after this step would further increase the purity of exosomes isolated.

3. Isolation of exosomes by ExoQuick-TC (EXOTC50A-1, SBI).
  ***Note:*** ExoQuick-TC from SBI is a proprietary polymer (PEG, polyethylene glycol) that gently precipitates exosomes between 30 and 200 nm in size from tissue culture media, urine, or spinal fluid. The detailed mechanism of this method can be found in SBI user manual: https://systembio.com/wp-content/uploads/MANUAL_EXOTCXXA-1-1.pdf.
  a. Transfer concentrated tumor culture supernatant to a sterile vessel and add 1/5 volume of ExoQuick-TC. Mix well by inverting the tube.
  b. Incubation the ExoQuick-TC/supernatant mixture 16–20 h at 4°C. The tubes should not be rotated or mixed during the incubation period and must remain upright.
    ***Note:*** Do not vortex or rotate the ExoQuick-TC/supernatant mixture, as it is recommended for PEG-based precipitation of exosomes.
  c. Centrifuge the ExoQuick-TC/supernatant mixture at 3,000 × *g* for 30 min. Centrifugation may be performed at either 25°C or 4°C with similar results. After centrifugation, exosomes may appear as a brown pellet at the bottom of vessel (Figure 1C).
  d. Discard the supernatant and centrifuge again at 3,000 × *g* for 5 min to remove the residual fluid.
  e. Resuspend the pellet with about 200 μL 1×PBS to make exosome solution.
  f. Exosome may be quantified by total protein quantification using BCA protein assay kit, or by EXOCET Exosome Quantitation Kit.

**Pause point:** Exosomes can be characterized as below and used immediately or stored at −80°C for 3 months.

***Note:*** Do not vortex but use pipette to handle exosome solution. Avoid repeatedly freeze and thaw exosomes. The diameter of exosomes ranges 40–60 nm accessed by dynamic light scattering (DLS) (Figure 1D) and the yield typically ranges 0.5–1 mg exosomes per 1×10^6^ cells based on BCA protein quantification method. Quality control assays of exosomes are routinely performed by DLS and BCA methods during −80°C storage. Within 3 months, the integrity, mean diameter, particle distribution, and concentration of exosomes remain nearly the same as fresh isolation.

4. Staining and imaging of exosome by transmission electron microscopy (TEM)
  a. Fix purified exosomes with 1 mL of 2% paraformaldehyde for 5 min.
  b. Glow-discharge the thin formvar/carbon film coated grids for 1 min.
  c. Load 5–7 μL exosome suspension (containing 50 μg exosomes) solution on the grid and incubate for 1 min.
    ***Note:*** If the concentration of exosome is too high, dilute it to 1/10–1/20.
  d. Stain with filtered 1% uranyl acetate solution on the surface of grid.
  e. Remove the excess uranyl acetate with filter paper.
  f. Rinse the grid with drop of water to remove the excess staining solution.
  g. Dry for 10 min at 25°C.
  h. Store grid in a box for future observation by TEM at 80 kV (Figure 5A).

5. Detection of markers of exosome by western blot (Figure 1E)
  a. Resuspend 0.2 mg exosome pellet in 200 μL RIPA buffer (with protease inhibitor added) and vortex 15 s.
  b. Place at ice for 5 min to allow complete lysis.
  c. Add 2×SDS buffer and heat at 95°C for 5 min. Chilled on ice before loading onto gel.
  d. Perform standard SDS-PAGE electrophoresis and western transfer onto PVDF.
  e. Block with 5% milk in Tris buffered saline + 0.05% Tween (TBST) for 1 h.
  f. Incubate blot 16–20 h at 4°C with exosome specific primary antibody at 1:1,000 dilution (5% milk in TBST).
  g. Wash 3 times with TBST.
  h. Incubate 1 h at 25°C with secondary antibody at 1:1,500 dilution (5% milk in TBST).
  i. Wash 3 times with TBST.
  j. Incubate blot with western blotting substrate and visualize on film.

***Note:*** It is important to note that not all cells produce exosomes with the same composition of tetraspanin markers, therefore not all the exosomal marker proteins (e.g., ALIX, TSG101, CD63, CD9, CD81, etc) could be definitely detected in your samples. However, it is strongly recommended to use some intracellular organelle markers to exclude contaminations of Golgi bodies (GM130), mitochondria (Cytochrome C) or nucleus (Histone H3). In our study, GM130, Cytochrome C, and Histone H3 was excluded to make sure the purity of exosomes(Yin et al., 2020).

### Generation of TDE-treated DCs *in vitro*

**Timing: 7 days**

**Figure 2:**
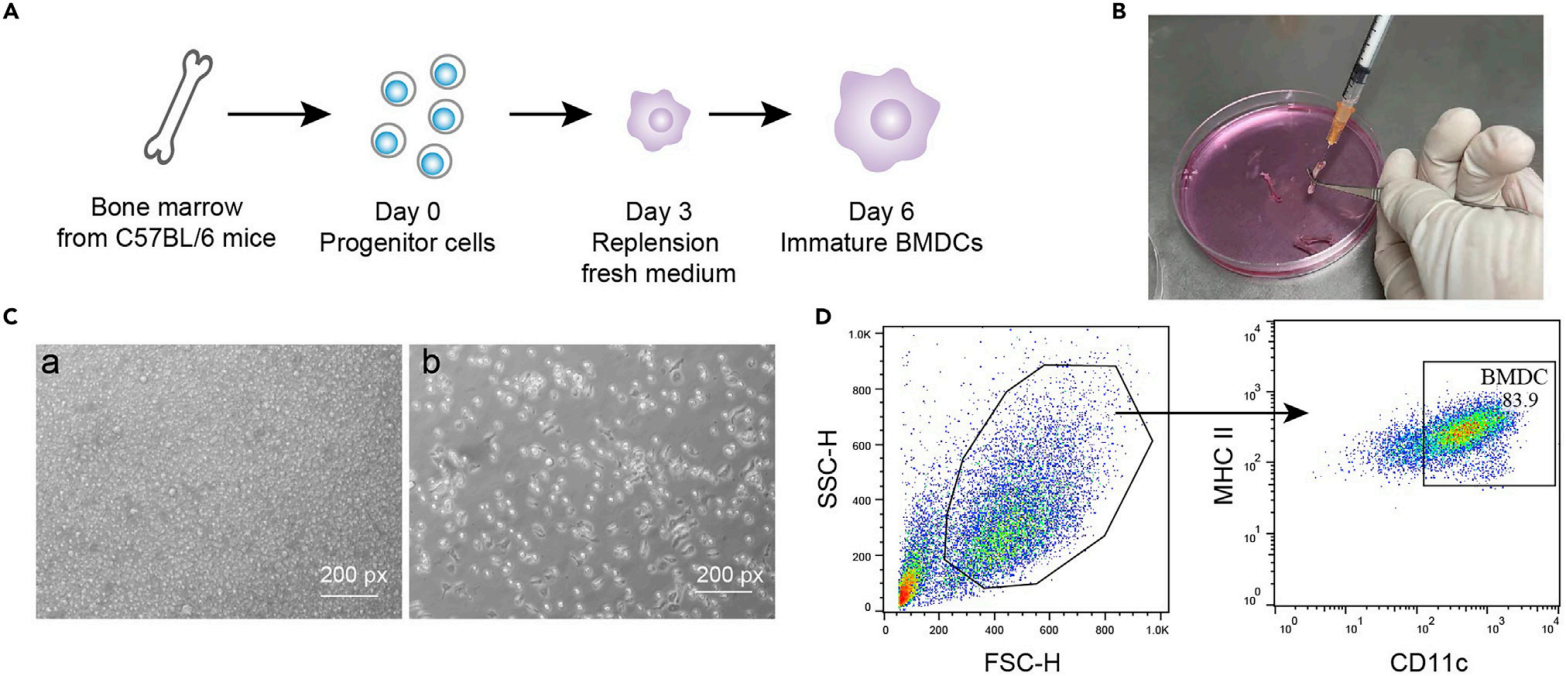
Generation of BMDC (A) Overview of BMDC generation. (B) Flush out the bone marrow into a 10 cm cell culture dish with a syringe plunge. (C) Morphology of BMDC on day 6 (a), and firmly adherent macrophages are remained (b). (D) Flow cytometry analysis of phenotype of BMDC (MHC II^+^CD11c^+^).

This step details how to generate bone marrow-derived dendritic cells (BMDCs) (Figure 2A).

6. Euthanize the C57BL/6 mice (about 8–10 weeks of age), spray the mice with 75% ethanol and fix the mice in a supine position.
7. Isolate the entire leg bones, carefully remove the connective tissue and muscle.
8. Gently cut down the condyles of leg bones.

***Note:*** Be careful not to break the bones.

9. Infuse the bone cavity with 1 mL RPMI medium (without FBS), and flush out the bone marrow into a 10 cm cell culture dish (Figure 2B).

***Note:*** Bone cavity can be flushed multiple times until all bone marrow is collected and the bones turn to white.

10. The cells from bone marrow are pass through a 70-μm cell strainer to prepare single-cell suspension, and centrifuge at 500–600 × *g* for 5 min.
11. Discard the supernatant, resuspend the cells in 2 mL of 1×ACK lysis buffer and incubate for 1 min at 25°C to eliminate red blood cells.
12. Add 20 mL of RPMI with 10% FBS to the cells and re-centrifuge at 500–600 × *g* for 5 min to terminate ACK lysis process.

***Note:*** After this step, use of lymphocyte gradient separation buffer is recommended to further ensure the purity of DCs.

13. After wash, resuspend and count the cells, then adjust the cell suspension to 3 × 10^5^ cells/mL with RPMI medium (10% FBS, 55 μM β-mercaptoethanol and 20 ng/mL rmGM-CSF plus 5 ng/mL rmIL-4).
14. Add 10 mL of cell suspension to a 10 cm cell culture dish and put in cell culture incubator.
15. Three days later, replenish 10 mL RPMI medium (10% FBS, 55 μM β-mercaptoethanol and 20 ng/mL rmGM-CSF plus 5 ng/mL rmIL-4) to the cells.
16. 6 days after differentiation, collect cells for further use.

***Note:*** When harvesting, only cells in suspension and loosely adherent cells should be isolated for further use and this protocol can render about 6–8 × 10^7^ DCs in sum.

17. Analyze the phenotype of DC by microscope and flow cytometry (MHC II^+^CD11c^+^) (Figures 2C and 2D).
  a. Discard the supernatant, and resuspend the cells with 50 μL Fc blocker buffer and mix well.
  b. Incubate the cells at 25°C for 5–10 min.
  c. Add 1/100 anti-mouse CD11c APC antibody and 1/200 anti-mouse MHC II FITC to each tube and mix well.
  d. Incubate the cells at 4°C for 20 min.
  e. After antibody incubation, neutralize the reaction by adding extra 1 mL FACS (1×PBS with 1% FBS) buffer in each tube.
  f. Centrifuge the cells at 500–600 × *g* for 5 min at 4°C, discard the supernatant, and resuspend the cells for FACS analysis with 1/1000 DAPI for separating live cells.
18. Treat BMDCs with 400 μg/mL TDEs for 48 h for further experiments.

### Generation of TDE-treated DCs *ex vivo*

**Timing: 48 h**

This step details how to label TDEs and isolate of TDE-treated DCs in lymph nodes (LNs).

**Figure 3:**
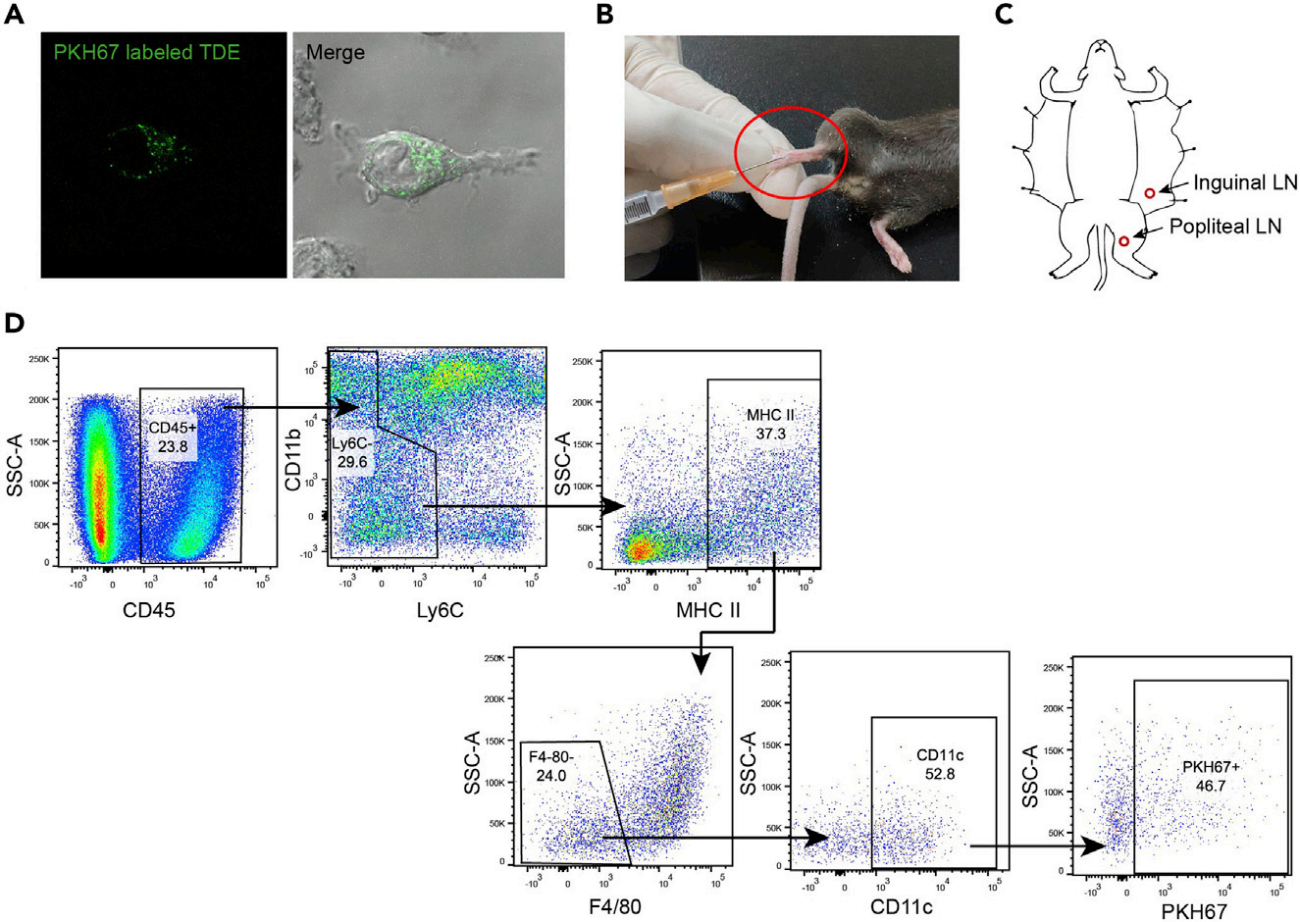
Isolation of TDE-treated DC *ex vivo* (A) Uptake of PKH67 labeled exosomes by BMDC. (B) Injection of PKH67 labeled exosomes into the mice footpad. (C) Location of popliteal lymph nodes and inguinal lymph nodes of mice. (D) Flow cytometry and gating strategy of PKH67-uptaken DCs.

19. Exosome labeling.
  a. Start with freshly isolated exosome pellets.
  b. Add 500 μL Diluent C from PKH67 kit to resuspend exosome pellets.
  c. Add 3 μL PKH67 dye into another 500 μL Diluent C tube.
  d. Mix b and c continuously at 25°C for 5 min.
  e. Neutralize the mixture by adding 1 mL 10% sterile BSA solution.
  f. Wash the PKH67 labeled exosomes 2 times with 1× sterile PBS.
  g. Collect the PKH67-labeled TDEs by ultracentrifugation (100,000 × *g*, 30 min, 25°C), and resuspend the exosomes in 1× sterile PBS.
  h. Treat the BMDCs with 400 μg/mL PKH67-labeled TDEs at 37°C for 6 h.
  i. Uptake of PKH67-labeled TDEs by BMDCs can be analyzed by Confocal microscope (Figure 3A).

**Pause point:** PKH67 labeled TDEs can be used immediately or stored at 4°C for 1 week.

20. Inject 1,000 μg PKH67 labeled TDEs (in 50 μL 1×PBS) into the footpad of C57BL/6 mice under tribromo-ethanol anesthesia (Figure 3B).

***Note:*** 0.5 mL tribromo-ethanol per mouse by intraperitoneal injection is used for anesthetization.

21. Isolate DCs in popliteal lymph nodes and inguinal lymph nodes at 48 h post injection (Figure 3C).
  a. Place a 70-μm cell strainer on top of a 50 mL centrifuge tube.
  b. Isolate the lymph nodes of mice, and put the lymph nodes in the cell strainer and grind with a syringe plunger, then flush the cells with 10 mL RPMI1640 (without FBS).
  c. Centrifuge the cell suspension at 500–600 × *g* for 5 min, and discard the supernatant.
  d. Resuspend the cell pellet with PBS (1% FBS) for staining.
  e. Discard the supernatant, and resuspend the cells with 50 μL Fc blocker buffer and mix well.
  f. Incubate the cells at 25°C for 5–10 min.
  g. Add antibody as “Antibody Mix Preparation-3” to each tube and mix well.
  h. Incubate the cells at 4°C for 20 min.
  i. After antibody incubation, neutralize the reaction by adding extra 1 mL FACS buffer in each tube.
  j. Centrifuge the cells at 500–600 × *g* for 5 min at 4°C, discard the supernatant, and resuspend the cells for FACS analysis with 1/1000 DAPI (0.1 μg/mL) for separating live cells.
  k. Collect the DCs (CD45^+^Ly6C^−^MHC II^+^F4/80^−^CD11c^+^PKH67^+^) by flow cytometer (Figure 3D) for further experiments.

***Note:*** B220 and CD14 is highly recommended to be included in the ex vivo isolation of DCs to further eliminate B220^+^ B cells and CD14^high^ monocytes.

### Isolation CD8^+^ T cells and CFSE labeling

**Timing: 70–90 min**

This step details how to anesthetize and sacrifice the mice, and prepare CFSE-labeled OTI CD8^+^ T cells.

**Figure 4:**
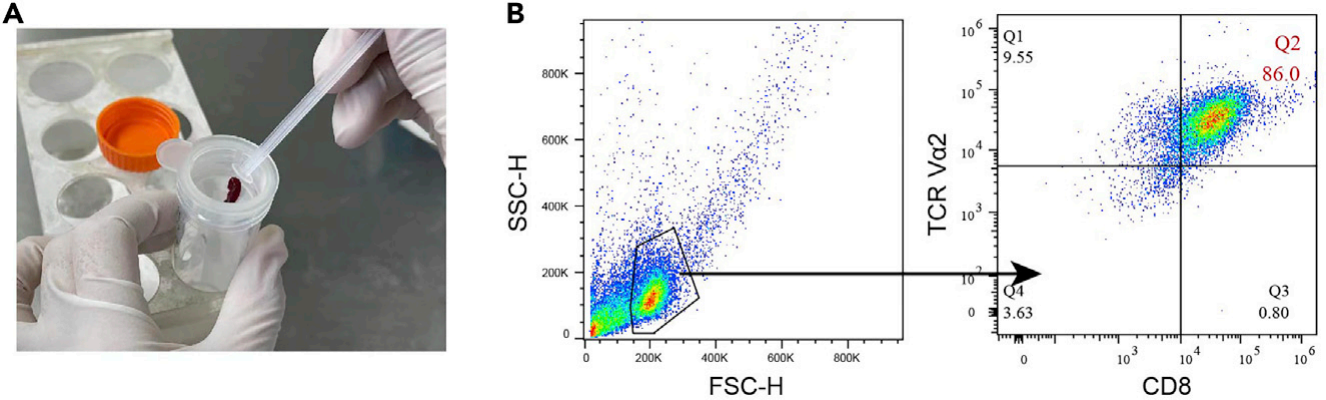
Isolation of CD8^+^ T cell (A) Grind the spleen on the 70 μm cell strainer with a syringe plunger. (B) Flow cytometry analysis of phenotype of OT I CD8^+^ T cell (TCR Vα 2^+^CD8^+^).
  c. Centrifuge the cell suspension at 500–600 × *g* for 5 min at 4°C once, and discard the supernatant.
  d. Resuspend the cells with 3 mL 1×RBC lysis buffer and let the process last for 3–5 min with intermittent swirling.
  e. Neutralize the RBC lysis process by adding 30 mL RPMI 1640 medium (or HBSS) (10 times volume of RBC lysis buffer added in the above step) and mix the cells well followed by passing through a 70 μm cell strainer again to exclude the cell aggregates or clots.
  f. Repeat step c and discard the supernatant to get splenocytes.
24. To isolate OT I CD8^+^ T cells
  a. Resuspend the splenocytes with 1×Mojo buffer to make sure the cell density is between 1×10^7^–1×10^8^ cells per mL (usually 0.4 mL buffer per spleen is sufficient based on our experience), and mix well by pipetting several times followed by transferring the cells into a new sterile 5 mL round bottom tube (BD Falcon).
  b. Add 10 μL Biotin-conjugated antibody cocktail per 100 μL (0.1 mL) cells (from spleen) and mix well. Then incubate the cells with antibodies on ice for 15 min.
  c. Add 10 μL nano-beads per 100 μL (0.1 mL) cells (the same volume used as the antibody cocktail). Then incubate the cells with antibodies on ice for 15 min.
  d. Add another 2 mL 1×Mojo buffer in the tube and put the tube in the magnate to separate CD8^+^ T cells from other cell compartments by standing on the bench for 5 min at 25°C.
  e. Hold the magnate to pour out the un-attached cells and collect them in a 15 mL conical centrifuge tube.
  f. Centrifuge the cell suspension at 500–600 × *g* for 5 min at 4°C and discard the supernatant. Generally, 1–2×10^7^ OT I CD8^+^ T cells can be isolated from a single mouse.
  g. FACS analyze the phenotype of isolated CD8^+^ OT I T cells (TCR Vα 2^+^CD8^+^) (Figure 4B).
  h. Discard the supernatant, and resuspend the cells with 50 μL Fc blocker buffer and mix well.
  i. Incubate the cells at 25°C for 5–10 min.
  j. Add 1/100 anti-mouse CD8a APC antibody and 1/100 anti-mouse TCRVa2 PE to each tube and mix well.
  k. Incubate the cells at 4°C for 20 min.
  l. After antibody incubation, neutralize the reaction by adding extra 1 mL FACS buffer in each tube.
  m. Centrifuge the cells at 500–600 × *g* for 5 min at 4°C, discard the supernatant, and resuspend the cells for FACS analysis with 1/1000 DAPI (0.1 μg/mL) for separating live cells.

***Note:*** it is recommended to leave 5%–10% of the cells as non-CFSE-labeled cells control for further FACS application.

22. Terminally anesthetize OTI transgenic mice by cervical dislocation.
23. Take out the spleen using tissue scissors and tissue forceps.
  a. Put the spleen on a 70 μm cell strainer on the top of a 50 mL centrifuge tube.
  b. Grind the spleen with a syringe plunger while flushing the strainer with 10 mL RPMI 1640 medium (without FBS) (Figure 4A).

25. CFSE labeling
  a. Resuspend the cells with 10 μM CFSE solution (diluted in 1×PBS) and mix well followed by incubation at 25°C for 10 min in the dark.
  b. After incubation, neutralize the CFSE labeling process by adding 0.2–0.5 mL FBS in the tube.
  c. Centrifuge the cells at 500–600 × *g* for 5 min at 4°C, and discard the supernatant.
  d. Resuspend the cells by RPMI 1640 medium added 10% FBS (BI) and 55 μM β-mercaptoethanol and count the cells.

### Co-culture to analyze T cell proliferation

**Timing: 6 days**

This step details the co-culture of TDE-treated DCs with CFSE-labeled OT I CD8^+^ T cells and how to analyze T cell proliferation.

**Figure 5:**
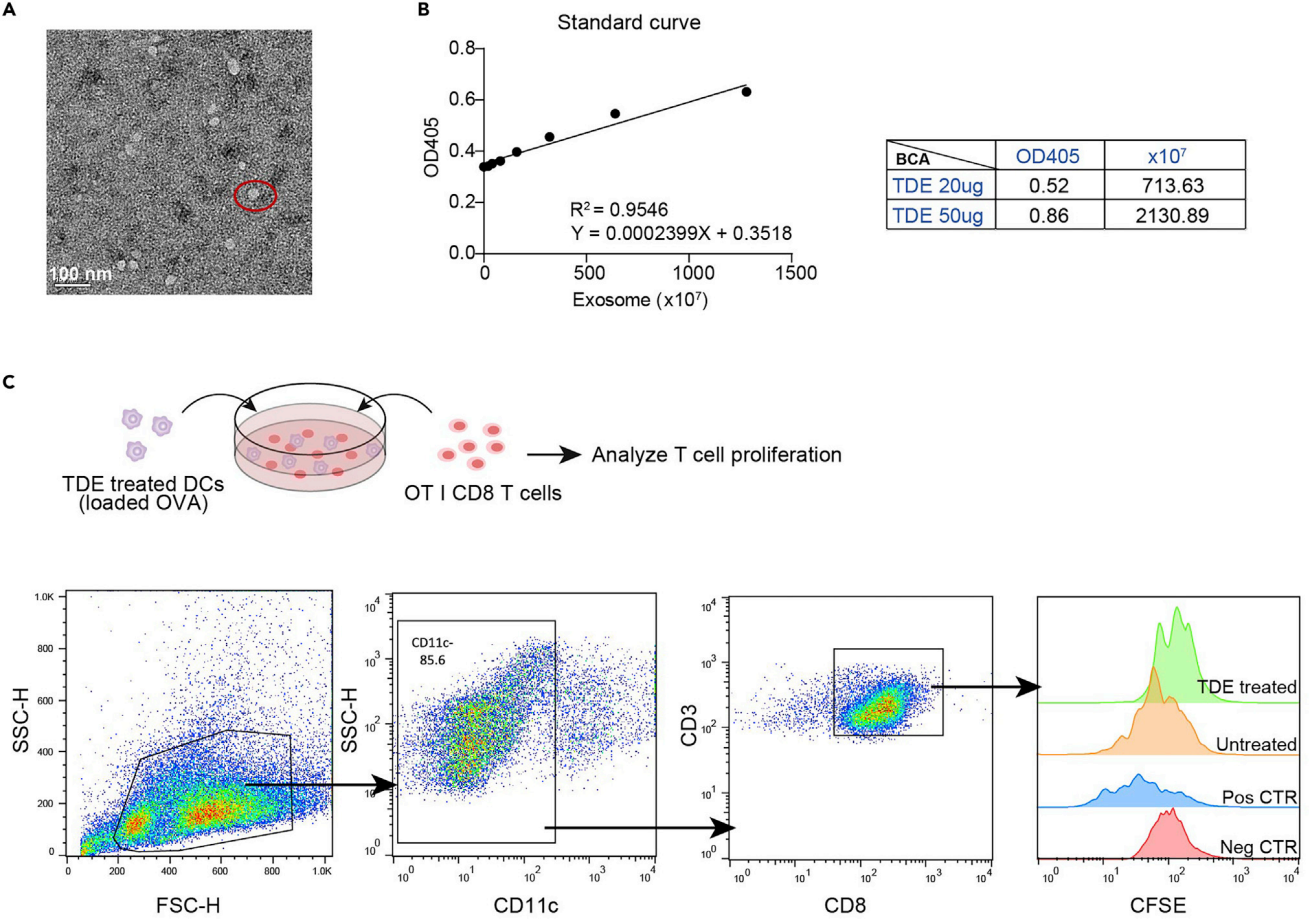
Immunosuppressive effect of TDE on DCs (A) Morphology of TDEs were analyzed by TEM. Scale bar, 100 nm. (B) Quantify exosomes by EXOCET Exosome Quantitation Kit and BCA method. (C) TDE-treated BMDCs or lymph node DCs, loaded with 2 mg/mL OVA, followed by antigen removal, and co-culture with OT I CD8^+^ T cells for 3 days. Proliferation was analyzed by flow cytometry. Neg CTR, undivided OT I CD8^+^ T cells. Pos CTR, OT I CD8^+^ T cell were treated with OVA_250-264_, as a positive control. Untreated, DCs without TDE treatment were co-cultured with OT I CD8^+^ T cells. TDE-treated, DCs with TDE treatment were co-cultured with OT I CD8^+^ T cells.

26. Collect TDE-treated DCs as described in step 18 and step 21, and treat the 1×10^6^ DCs with 2 mg/mL OVA antigen for 48 h (applicable for TDE-treated DCs both from *in vitro* or *ex vivo*).
27. After treatment, collect and wash the cells by centrifuging at 500–600 × *g* for 5 min at 4°C to eliminate residual OVA antigen.
28. Discard the supernatant and resuspend the cells with proper volume (200~400 μL) of 1640 medium added 10% FBS (BI) and 55 μM β-mercaptoethanol (Gibco) and count the DCs.
29. Co-culture the DCs (step 27) and CFSE-labeled OTI CD8^+^ T cells (step 24) at the ratio between 1:5 to 1:8.
30. 4 days later collect and centrifuge the cells at 500–600 × *g* for 5 min at 4°C.

31. FACS analyze the proliferation of OT I CD8^+^ T cells (Figure 5C).
  a. Discard the supernatant, and resuspend the cells with 50 μL Fc blocker buffer and mix well.
  b. Incubate the cells at 25°C for 5–10 min.
  c. Add 1/100 anti-mouse CD8 APC antibody, 1/100 anti-mouse CD11c PE and 1/200 anti-mouse CD3 PE/Cy7 to each tube and mix well.
    ***Note:*** Staining with CD11c can exclude CD8^+^ DCs from CD8^+^ T cells.
  d. Incubate the cells at 4°C for 20 min.
  e. After antibody incubation, neutralize the reaction by adding extra 1 mL FACS buffer in each tube.
  f. Centrifuge the cells at 500–600 × *g* for 5 min at 4°C, discard the supernatant, and resuspend the cells for FACS analysis.
  g. CFSE^low^ CD11c^−^CD3^+^CD8^+^ population is considered as proliferating OT I CD8^+^ T cells.

## Expected outcomes

Following this protocol, we isolated exosomes from tumor culture medium, and evaluate the immunosuppressive function of tumor-derived exosomes on DC *in vitro* and *ex vivo*.

Morphologies of the purified exosomes were characterized by transmission electron microscopy (TEM; Figure 5A), showing a mean diameter of ~40 nm, consistent with the size of previous reports (Kalluri, 2016; Kalluri and LeBleu, 2020). Western blot analysis revealed the presence of exosome-enriched proteins (e.g., ALIX, HSP70, TSG101, CD9, and CD81)(Hessvik and Llorente, 2017; Huang-Doran et al., 2017)(Data as shown in (Yin et al., 2020)). In our experience, we also calculated the amount of exosomes by EXOCET Exosome Quantitation Kit and found that the number of exosomes were highly correlated with the protein content by BCA (Figure 5B). Thus, these two methods may be used to quantify exosome.

Isolated TDEs were used to treat BMDCs *in vitro* or lymph node DCs *ex vivo*, then we analyzed proliferation of OT I CD8^+^ T cell to identify the function of TDE-treated DCs. As the data shown, compared with untreated DCs, TDE-treated DCs had a defective T cell priming capability (Figure 5C). The dysfunctional DCs was the result of the uptake of TDE-derived FAs. For full details, we refer to (Yin et al., 2020).

## Troubleshooting

### Problem 1

Low yield and low purity of BMDCs.

### Potential solution

Increase the number of leg bones used to differentiate BMDCs according to your experiment needs. And the purity of GM-CSF differentiated BMDC is strongly recommended to be tested every time before use, since low purity of BMDCs may lead to inaccurate experiment outcomes.

### Problem 2

The effect of tumor-derived exosomes (TDEs) on BMDCs is unreproducible.

### Potential solution

If you find (in some rare cases) TDEs-treated BMDCs failed to or are less able to induce evident T cell suppression. You could

1. Re-extract exosomes using fresh extraction kit/reagent.
2. Check the status of parent tumor cells.
3. Make sure exosome-depleted FBS is used during exosomes collection.
4. Do the quality control of TDEs every time before use to make sure the isolation of TDEs is successful by performing TEM or WB assays described in steps 4 and 5.

### Possible reasons

Based on our experience, exosomes from different parent cells may have different impact on DC function. Exosomes can be taken as an important FFAs carrier. Thus, the quality and species of FFAs encapsulated in exosome may vary due to the type and status of parent cells (Haraszti et al., 2016). For example, FFAs content in non-cancerous cells (NIH-3T3) derived exosomes is far less than that in tumor cells derived exosomes (Yin et al., 2020).

### Problem 3

CFSE labeling associated toxicity

### Potential solution

Resuspend purified CD8^+^ T cells with 10 μM ready-to-use CFSE solution (in 1× PBS), rather than adding 10 mM CFSE stock solution in suspended CD8^+^ T cells, can avoid CFSE labeling associated toxicity to a great extent. And 10 min incubation time at 25°C is enough, while exceeding the incubation time may increase T cell death.

## Resource availability

### Lead contact

Further information and requests for resources and reagents should be directed to and will be fulfilled by the lead contact, Lingtao Jin (LJIN1@ufl.edu).

### Materials availability

This study did not generate any unique materials or reagents.

### Data and code availability

This study did not generate new unique datasets or code.

## References

[bib1] Haraszti R.A., Didiot M.C., Sapp E., Leszyk J., Shaffer S.A., Rockwell H.E., Gao F., Narain N.R., DiFiglia M., Kiebish M.A. (2016). High-resolution proteomic and lipidomic analysis of exosomes and microvesicles from different cell sources. J Extracell Vesicles.

[bib2] Hessvik N.P., Llorente A. (2017). Current knowledge on exosome biogenesis and release. Cell Mol Life Sci.

[bib3] Huang-Doran I., Zhang C.Y., Vidal-Puig A. (2017). Extracellular vesicles: novel mediators of cell communication in metabolic disease. Trends Endocrinol Metab.

[bib4] Kalluri R. (2016). The biology and function of exosomes in cancer. J Clin Invest.

[bib5] Kalluri R., LeBleu V.S. (2020). The biology, function, and biomedical applications of exosomes. Science.

[bib6] Yin X., Zeng W., Wu B., Wang L., Wang Z., Tian H., Wang L., Jiang Y., Clay R., Wei X. (2020). PPARalpha inhibition overcomes tumor-derived exosomal lipid-induced dendritic cell dysfunction. Cell Rep.

